# Supplementary material for: In Silico Predicting the Presence of the S100B Motif in Edible Plants and Detecting Its Immunoreactive Materials: Perspectives for Functional Foods, Dietary Supplements and Phytotherapies
Source: Int J Mol Sci. 2024 Sep 11;25(18):9813. doi: 10.3390/ijms25189813 (PMC11431829; doi:10.3390/ijms25189813)
Supplement: Supplementary file 1 [file ijms-25-09813-s001.zip › ijms-3146001-Figure S1.pdf]

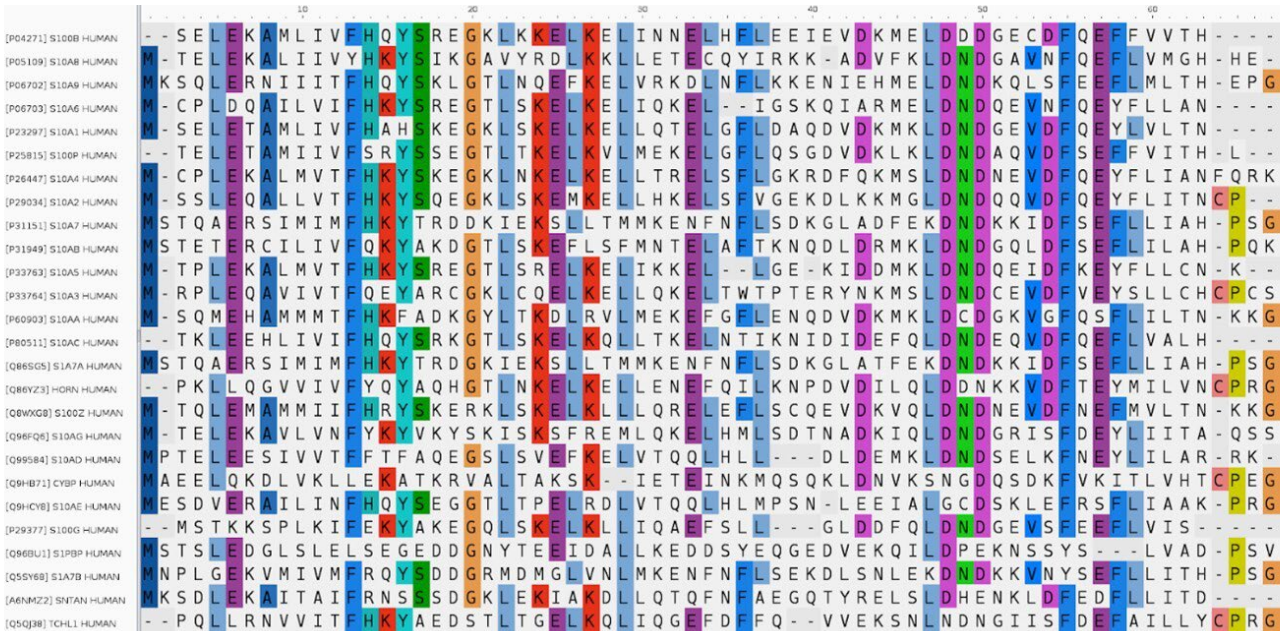

Figure S1. The conserved domains showing significant structural homology with the benchmark protein S100B were identified and separated out, reporting the alignment with other members of the S100 family and showing the specificity of S10B as the sequence we used for in silico and immunological test. Sequence alignment of S100B with other S100 protein family members.
