# Supplementary material for: In Silico Predicting the Presence of the S100B Motif in Edible Plants and Detecting Its Immunoreactive Materials: Perspectives for Functional Foods, Dietary Supplements and Phytotherapies
Source: Int J Mol Sci. 2024 Sep 11;25(18):9813. doi: 10.3390/ijms25189813 (PMC11431829; doi:10.3390/ijms25189813)
Supplement: Supplementary file 1 [file ijms-25-09813-s001.zip › ijms-3146001-Table S1.pdf]

Table S1. Sample description.

Sample	Specimen	Quantity (mg)	Origin
Açai	Berries powder	119	Organic açai berries from Brazil. "NaturaleBio"- International Food Europe srl; Rome, Italy
Banana	Pulp powder	110	Fruit from Ecuador. "Purasana"- Biovita bvba Heulestraat; Gullegem, Belgium
Banana	Fresh pulp	80	ns
Baobab	Fruit pulp powder	153	Raw baobab from South Africa. "Cibocrudo srl"; Ciciliano, Italy
Broccoli	Fresh inflorescences	83	Sicily, Italy
Cabbage	Fresh leaves	84	ns, Italy
Cocoa	Fava beans powder	110	Organic fairtrade cacao from UK "Naturya" Bath, BA2 7PQ, UK
Durian	Fresh pulp	122	Thailand
Durian	Freeze/Dry pulp	105	"Sunshine international Co.ltd" Songpinong Thamai, Chanthaburi 22120, Thailand
Durian	Pulp powder	80	Thailand
Graviola	Fruit and plant extract powder 4:1	144	(Graviola from Perù) "AMTEC Trading GmbH"; Wolfsberg, Austria
Jack fruit	Lyophilized pulp	139	Fruit from Tanzania. "Cibocrudo srl"; Ciciliano, Italy
Kiwi	Fresh pulp	100	ns, Italy
Kombucha	Lyophilized tea powder	128	Fermented tea from China. "Xi' an Le Sen Bio-technology Co. Ltd"; Xi'an, China
Laurel	Fresh leaves	106	ns, Italy
Mela Annurca (*cps*)	Apple Annurca dry extract	133	Apple from Campania, Italy "4TIFY" – GeneS; Rome, Italy

Reishi Mushroom	Powder	102	"Terra elements GmbH"; München, Germany
Sage	Fresh leaves	103	ns, Italy
Salad	Fresh leaves	86	ns, Italy
Spinach	Powder	104	Spinach leaves from Germany. "Raab Vitalfood GmbH"; Rohrbach, Germany
Spinach	Fresh leaves	85	ns, Italy
Sunflower	Seeds powder	133	Sunflower seeds from Austria. "Schalk Mühle GmbH & Co KG"; Ilz, Austria

Abbreviations: ns = not specified
